# Fenfluramine: New Treatment for Seizures in Dravet Syndrome

**DOI:** 10.15844/pedneurbriefs-34-8

**Published:** 2020-03-12

**Authors:** Joanna Garcia Pierce, Divakar S. Mithal

**Affiliations:** 1 Division of Neurology, Ann & Robert H. Lurie Children’s Hospital of Chicago, Chicago, IL; 2 Departments of Pediatrics and Neurology, Northwestern University Feinberg School of Medicine, Chicago, IL

**Keywords:** Dravet Syndrome, Seizures, Fenfluramine

## Abstract

Investigators for the FAiRE DS Study Group assessed the efficacy and safety of Fenfluramine for treating seizures in patients less than 18 y.o. with Dravet Syndrome in an international double-blind, placebo-controlled clinical trial. A total of 119 patients (mean age 9.0 y, 54% male) were enrolled in the study.

Investigators for the FAiRE DS Study Group assessed the efficacy and safety of Fenfluramine for treating seizures in patients less than 18 y.o. with Dravet Syndrome in an international double-blind, placebo-controlled clinical trial. A total of 119 patients (mean age 9.0 y, 54% male) were enrolled in the study. Patients were on stable anti-epileptic drugs with poorly controlled convulsive seizures, with an average monthly convulsive seizure frequency (MCSF) of 40.3 in the prior 28 days. For a total of 14 weeks, caregivers provided participants with either placebo, Fenfluramine 0.2mg/kg/day or Fenfluramine 0.7mg/kg/day. Exclusion criteria included recent use of Stiripentol, Cannabidiol or Serotonergic medications. Importantly, patients were monitored with echocardiograms and electrocardiograms.

The study met the primary endpoint as patients saw a significant estimated decline in the MCSF relative to placebo for both Fenfluramine 0.7mg/kg/day (62.3% reduction, p>0.0001) and 0.2mg/kg/day (32.4% reduction, p=0.0209). For the higher dose of Fenfluramine, a number of secondary endpoints were met, including reduction in rescue medication use, improvements in both caregiver and investigator assessments and improvement of some behavioral measures. The high dose of Fenfluramine resulted in weight loss for patients aged 13-18 years. Adverse side-effects where reported more in both Fenfluramine groups (95%) compared to the placebo group (65%). The most common side effects were decreased appetite, diarrhea, nasopharyngitis, lethargy, and pyrexia. Fenfluramine was not associated with any cardiovascular complications. [1]

COMMENTARY. Fenfluramine is an amphetamine derivative that was found to have anti-epileptic effects since 1980s [2]. The medication became popular in the 1990s as an appetite suppressant but was removed from the market due to cardiovascular complications at high doses. The mechanism by which Fenfluramine treats seizures is believed to be through regulation of serotonin signaling. Invertebrate animal models with SCN1A mutations demonstrate activity at 5-HT1D and 5-HT2C receptors [3].

Dravet Syndrome is an epileptic encephalopathy with a significant seizure burden, often refractory to anticonvulsant treatment. In recent years, Stiripentol and Cannabidiol have been approved for the treatment of seizures in Dravet Syndrome, both having encouraging clinical trial and post-approval data [4]. In this study 49% of patients were previously on Stiripentol and 26% were previously on Cannabidiol, indicating an ongoing need for additional treatment options.

The paper demonstrates that low dose Fenfluramine is a safe and effective add-on medication for providers to consider to significantly reduce seizure frequency in patients with Dravet Syndrome. Importantly, despite extensive monitoring, no cardiovascular risk was associated with either dose of Fenfluramine. Weight loss was a mild side effect that should be well-tolerated if a patient experiences seizure reduction. Furthermore, a separate retrospective analysis of 10 patients with Dravet Syndrome on long-term Fenfluramine (6-27 years) demonstrated better seizure control without any significant medication side effects [5]. The promising results in this study of Fenfluramine in Dravet Syndrome raise the possibility of future studies using the medication to treat additional forms of epilepsy.

## Disclosures

The authors have declared that no competing interests exist.
